# Interplay between personality traits and learning strategies: the missing link

**DOI:** 10.1152/advan.00001.2022

**Published:** 2022-09-22

**Authors:** Read A. Albar, Ayman M. A. Mohamed, Mohieddin A. B. Albarazi, Sean McAleer, Hassan S. Shaibah

**Affiliations:** ^1^College of Medicine, Alfaisal University, Riyadh, Saudi Arabia; ^2^Centre for Medical Education, University of Dundee, Dundee, United Kingdom

**Keywords:** individualized support, learning strategies, mentorship, personality

## Abstract

Students with varying personality traits are likely to employ diverse learning and study strategies. However, this relationship has never been explored in the medical education context. This study’s aim was to explore the relationship between learning strategies and personality traits among medical students. This study was a cross-sectional study, and a quantitative approach was employed using two self-administered questionnaires: one to assess the personality traits from the Five-Factor Model (Conscientiousness, Neuroticism, Extraversion, Openness, and Agreeableness), and the other to assess 10 learning strategies (Anxiety, Attitude, Concentration, Information Processing, Motivation, Selecting Main Ideas, Self-Testing, Test Strategies, Time Management, and Using Academic Resources). A stratified random sampling technique was used to recruit medical students at Alfaisal University in the preclinical and clinical years (*N* = 309). Pearson correlation coefficient was used to measure the relationship between variables, and linear regression was used to evaluate how personality traits predicted learning strategy selection. Personality traits predicted the selection of learning strategies, especially Conscientiousness and Neuroticism. Conscientiousness showed a positive correlation with seven learning strategies and was the most important predictor of learning strategies students employ. Neuroticism correlations and predictions were negative. The other three traits showed weaker correlations. These correlations were between Extraversion and Using Academic Resources (*r* = 0.27), Information Processing (*r* = 0.23), and Attitude (*r* = 0.19); Openness and Information Processing (*r* = 0.29); and Agreeableness and Attitude (*r* = 0.29). All personality domains influence at least one learning strategy, especially Conscientiousness and Neuroticism. This study helps build a foundation for individualized coaching and mentorship in medical education.

**NEW & NOTEWORTHY** This study aspires to build a foundation for individualized coaching and mentorship in medical education through utilizing personality traits to empower academic success. We demonstrate that all personality domains influence students’ selection of at least one learning strategy, especially Conscientiousness and Neuroticism.

## INTRODUCTION

Medical schools impart a tremendous amount of academic material within a limited period of time (1). Students’ success in handling such a workload is greatly influenced by their personalities, which are vital components of students’ characteristic traits (2), as well as by the learning strategies they employ, which are crucial components of self-regulated learning (SRL) strategies (3–6).

Students who are viewed as self-regulated learners proactively engage in the learning process behaviorally, emotionally, motivationally, cognitively, and metacognitively (7, 8). SRL strategies are constructive processes where students self-activate, direct, and regulate their efforts to obtain knowledge and develop skills by employing specific learning and study strategies. They also monitor and assess their progress throughout the various phases of the learning process (9).

**Figure 1.**
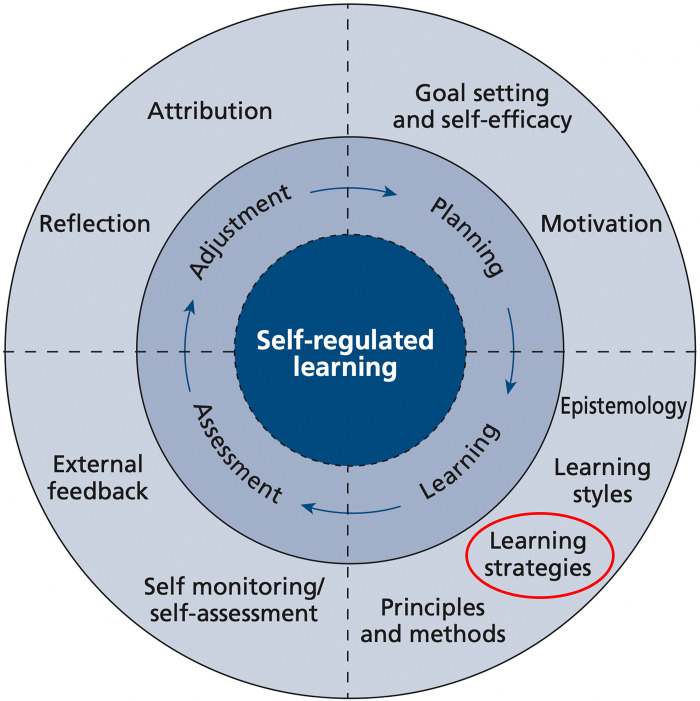
Self-regulated learning in medical education. Adapted from Ref. 6, with permission from Wiley.

Medical educators adapted the “SRL model” from studies in educational psychology (6). This model reflects four steps of a continuous cycle (planning, learning, assessment, and adjustment) and the underlying elements within each of these steps (Fig. 1). It presents a multifaceted and intersecting network that underlies SRL strategies, including personality and cognition development.

Fortunately, SRL strategies are composed of skills that can be learned, and medical educators play a vital role in assisting students to develop such skills, which are needed for succeeding academically and professionally (10). According to the aforementioned SRL model, learning strategies are vital elements of the learning step (Fig. 1).

Learning strategies are one element in the SRL model (Fig. 1). They are methods of approaching a learning task through thoughts, behaviors, attitudes, motivation, and beliefs (11). They can also be referred to as a set of skills that facilitate performance on a task and assist students in processing, organizing, and managing information according to academic requirements, task features, and specific contexts (12). Beckman (13) illustrated the crucial effects of learning strategies on students’ success. He suggested that learning and study strategies empower students to become more literate, productive, responsible, independent, and assured in their thoughts. Moreover, students are able to recognize and rectify their mistakes through developing their learning processes.

With this in mind, understanding how students learn is vital (14). The empirical literature suggests that learning strategies are not the only factors that influence learning outcomes. Students’ characteristics, such as personality traits, also have the potential to influence learning outcomes (15). Additionally, students’ personalities have been thought of as the basis for understanding individual differences in learning (16), which can help faculty better individualize mentorship and identify students at academic risk.

Personality scientists show subtle differences in their definition of personality, which reflect their different theoretical understanding and beliefs about the term. However, almost all have employed the following definition: personality traits are “psychological qualities that contribute to an individual’s enduring and distinctive patterns of feelings, thinking, and behaving” (17). Thus, personality traits, which are consistently expressed over time, strongly influence one’s expectations, self-perceptions, values, and attitudes (18, 19). Therefore, personality traits account for individual differences in learning (20), which make them equally important as cognitive abilities for thriving in medical studies and the medical profession (21). With this in mind, Vygotsky (22) emphasized that to understand where students are going (i.e., acquiring necessary skills to succeed), educators need to understand where these students are coming from as well (i.e., students’ characteristics).

One of the most researched personality theories was based on trait approach and was often called the trait (or dispositional) theory. This trait perspective revolves around the identification of traits, descriptions of typical styles of experience and behavior, and measurement of such traits (broad or specific) that make up one’s personality (23). Trait-approach psychologists consider traits to be the major units of personality, and by understanding them they believe they can better grasp the differences between individuals.

One of the robust models that has emerged to assess major personality traits is the Five-Factor Model (FFM) (24–26). It is based on the lexical hypothesis, which proposes that the significant aspects of people’s life are encoded into the natural-language lexicon; that is, many descriptors of personality traits exist within natural languages (27, 28). That is why the FFM was developed empirically rather than theoretically and was founded through psycho-lexical analysis or factor analysis (29) of thousands of English words, describing and correlating personality traits (30, 31). These factors are Openness to Experience, Conscientiousness, Extraversion, Agreeableness, and Neuroticism. (Table 1).

**Table 1.** Description of personality traits

Traits	Description
Openness to experience	Refers to the person’s tendency to be imaginative, intellectually curious, unconventional, and fond of variety.
Conscientiousness	Concerned with impulse control and an individual’s tendency to be responsible, purposeful, organized, and determined.
Extraversion	Refers to someone’s tendency to be sociable, energetic, talkative, and assertive, and to experience positive emotions (e.g., happiness) and/or thoughts (e.g., optimism).
Agreeableness	Concerned with how well a person gets along with others and the tendency to be altruistic, cooperative, sympathetic, trusting, and willing to help others.
Neuroticism	Refers to the individual’s inclination to experience negative emotions such as anxiety, sadness, and hostility, and negative thoughts such as self-doubt and inability to control cravings and urges. It is considered opposite to emotional stability.

The acronym “OCEAN” was created to represent the factors listed. It is worth noting that the 5 factors fall on a spectrum and are not considered as discrete categories. That is, a person can score high, low, or in between on each scale (e.g., high in Extraversion, low in Neuroticism, average in Agreeableness). This continuum perspective allows more flexibility in viewing personalities rather than boxing individuals into different types.

In medical education, personality traits have previously been studied to identify which traits affect academic performance (21). On the other hand, studies on learning strategies were also mainly focused on academic performance (32–35). However, the correlation between personality traits and selection of learning strategies is limited within the medical education context. Thus, the purpose of this study was to explore whether personality traits influence the selection of learning strategies among medical students. The findings could be used to help individualize mentorship and enhance academic counseling in medical schools.

## METHODS

The target population was composed of male and female undergraduate medical students from year 1 to year 5 enrolled at the College of Medicine at Alfaisal University (AU), Riyadh, Saudi Arabia (*n* = 1,140). At Alfaisal, the first 3 years are preclinical and the last 2 years are the clerkship years. Students in the University Preparatory Program and Internship (clinical training year) were excluded.

### Study Design

A case study approach was used to identify the interactions between personality traits and learning strategies and was informed by educational action research principles. This project was cross-sectional, and a quantitative approach was employed.

### The Sampling Method

A stratified random sampling was used to produce a better representation of the population and minimize the chance of selection biases and sampling errors.

With an online sample size calculator (https://surveysystem.com/sscalc.htm), the sample size was estimated to be 288, with confidence level of 95% and confidence interval (margin of error) of 5%. Participants were required to complete both personality traits [NEO-Five Factor Inventory-3 (NEO-FFI-3)] and learning strategies [Learning and Study Strategies Inventory (LASSI)] questionnaires.

### Recruitment Strategy

Students were divided into preclinical (1st, 2nd, and 3rd) and clinical (4th and 5th) years. Randomizer.org was used to randomly select the students from the student list in each year. The recruitment was divided into two phases: 1st phase for preclinical years and 2nd phase for clinical years, with 2 wk between the two phases.

In the preclinical year phase, 250 students were selected to participate. In the clinical year phase, 120 students were selected. An e-mail explaining the project and containing the informed consent form was sent to the selected students. Students who signed the consent form were e-mailed two links containing both questionnaires (NEO-FFI-3 and LASSI). A maximum of three e-mail reminders were sent to students who received the links but did not complete any of the questionnaires. After 2 wk of the third reminder, such students who consented but did not complete the questionnaires were excluded. A second cycle of random selection of students was conducted to compensate for the students who did not sign the consent after two e-mail reminders.

### The Questionnaires

#### The Learning and Study Strategies Inventory.

Learning strategies were assessed by administering the Learning and Study Strategies Inventory (LASSI), third edition (11). It is a 10-scale, 60-item assessment of students’ awareness about and use of learning strategies related to Skill (Information Processing, Selecting Main Ideas, and Test Strategies), Will (Anxiety, Attitude, and Motivation), and Self-Regulation (Concentration, Self-Testing, Time Management, and Using Academic Resources). It utilizes a five-point Likert scale (1 = Not at all typical of me to 5 = Very much typical of me). The LASSI provides standardized scores (percentile score equivalents) based on normative samples for the 10 scales included with the instrument.

All scoring, reporting, and graphics are generated automatically, and students receive a feedback report immediately after completing the questionnaire. These strategies are outlined in Table 2.

**Table 2.** Description of learning strategies measured by LASSI

Components (Latent Constructs)	Learning and Study Strategies	Description
Skill	Information Processing (IFP)	Represents the ability of students to bridge their previous knowledge to what they want to learn or remember to produce meaningful learning.
	Selecting Main Ideas (SMI)	Represents the skills to identify key information in study material and differentiate it from supporting details.
	Test Strategies (TST)	Implies students’ ability to properly prepare for tests of different types and items.
Will	Anxiety (ANX)*	Represents the tendency of students to worry about their academic performance in ways that disturb their concentration even when they are well prepared.
	Attitude (ATT)	Implies students’ interests in achieving academic success and their beliefs of the worthiness in attending college and obtaining a degree.
	Motivation (MOT)	Represents students’ willingness to take responsibility and exert the necessary efforts to attain academic goals.
Self-Regulation	Concentration (CON)	Implies students’ ability to direct and maintain their focus toward academic tasks.
	Self-Testing (SFT)	Represents students’ metacognitive skills such as creating potential questions that they might be asked about on a test and their monitoring skills such as periodic reviews of content.
	Time Management (TMT)	Implies the tendency to strategically managing time in order to complete academic tasks in a timely manner.
	Using Academic Resources (UAR)	Represents students’ willingness to seek guidance and utilize various academic resources, especially when they face academic difficulties.

* ANX is the only strategy that is scored in reverse. That is, a higher score in the Learning and Study Strategies Inventory (LASSI) means less anxiety.

The LASSI was chosen because it is both a diagnostic tool that identifies students’ strengths and weaknesses and a prescriptive tool that provides feedback to improve academic achievement at high school and university levels (36), which in turn can be of benefit for mentoring students.

#### The NEO-Five Factor Inventory-3.

The test consists of 60 items that measure the five dimensions of personality, i.e., Openness, Conscientiousness, Extraversion, Agreeableness, and Neuroticism (Table 1*)*. It utilizes a Likert-type scale (1= strongly disagree, 5 = strongly agree). The NEO-FFI-3 provides standardized scores (T scores) based on different normative samples for the five personality domains included with the instrument. Based on the total T scores, students are categorized in each trait into the following scales: very low (<35), low (35–44), average (45–55), high (56–65), and very high (>65). All scoring, reporting, and graphics are generated automatically, and students receive the feedback report after completing the questionnaire.

It is the most widely used measurement for traits according to the Five-Factor Model (37) and provides a general description of normal personality traits relevant to clinical, counseling, and educational situations as well as providing a detailed report about each of the five major personality traits.

### Quantitative Analysis

Descriptive statistics were performed for sex, preclinical/clinical years, and the five T scores derived from NEO-FFI-3 (Openness, Conscientiousness, Extraversion, Agreeableness, and Neuroticism) and the 10 LASSI scores (Anxiety, Attitude, Concentration, Information Processing, Motivation, Selecting Main Ideas, Self-Testing, Test Strategies, Time Management, and Using Academic Resources).

Pearson correlation was utilized to study the correlation between T scores of the five traits and the 10 strategies. Pearson correlation ≥ 0.3 with significant *P* value was considered a potentially meaningful correlation in line with commonly accepted correlation ranges found in psychological studies, especially personality-based studies (38).

To determine the predictive value of the 5 independent variables (personality traits) on the 10 dependent variables (learning strategies), a multiple linear regression model was used and adjusted for the potential confounders including sex and preclinical/clinical years.

All statistical analyses were performed by using SAS Survey Procedures (SAS 9.4; SAS Institute Inc, Cary, NC). Statistical significance was defined by the two-tailed test with a *P* value < 0.05.

This study was approved by the Institutional Review Board (IRB) at Alfaisal University (IRB-18045).

## RESULTS

There were 309 participants included in this study, and the average age was 21 yr. Sixty-nine percent were female (*n* = 213), and nearly half were either in their first year (*n* = 73, 23.6%) or second year (*n* = 80, 25.9%)

### Pearson Correlation Coefficient Analysis

To investigate the linear relationship between the T scores of the five personality traits and the 10 LASSI scores, a Pearson correlation coefficient analysis was performed (Table 3). Based on Cohen’s criteria (39), the potentially meaningful linear correlation was set at ≥0.3. Conscientiousness T scores were correlated with seven LASSI scores, and the highest correlation was Motivation (*r* = 0.68), followed by Time Management (*r* = 0.64), Concentration (*r* = 0.56), Test Strategies (*r* = 0.41), Attitude (*r* = 0.34), Selecting Main Ideas (*r* = 0.32), and Self-Testing (*r* = 0.32). Moreover, Neuroticism T scores were correlated with four LASSI scores, and the highest correlation was Anxiety (*r* = −0.56), followed by Test Strategies (r = −0.39), Selecting Main Ideas (*r* = −0.33), and Concentration (*r* = −0.32). Although the *P* value was significant for the other three T scores (Openness, Extraversion, and Agreeableness) and a few LASSI scores, the Pearson correlation coefficient was <0.3. Those correlations were not considered as potentially meaningful based on Cohen’s criteria. It is worth noting that Anxiety was the only scale that had reverse scoring (Table 3).

**Table 3.** Pearson correlation coefficient among personality domains and LASSI scores

	ANX	ATT	CON	INP	MOT	SMI	SFT	TST	TMT	UAR
Openness										
Correlation coefficient	0.07	0.02	−0.03	0.29‡	−0.10	0.11*	0.10	0.10	−0.11	0.01
*P* value	0.21	0.80	0.57	<0.001	0.36	0.05	0.07	0.22	0.07	0.87
Conscientiousness										
Correlation coefficient	0.18†	0.34‡	0.56‡	0.24‡	0.68‡	0.32‡	0.32‡	0.41‡	0.64‡	0.11
*P* value	0.002	<0.001	<0.001	<0.001	<0.0001	<0.001	<0.001	<0.001	<0.001	0.06
Extraversion										
Correlation coefficient	0.14*	0.19†	0.10	0.23‡	0.04	0.14*	0.09	0.07	0.08	0.27‡
*P* value	0.012	0.001	0.07	<0.001	0.54	0.012	0.14	0.20	0.18	<0.001
Agreeableness										
Correlation coefficient	0.06	0.29‡	0.05	0.01	0.00	0.06	0.01	0.07	−0.06	0.03
*P* value	0.32	<0.001	0.37	0.88	0.98	0.28	0.85	0.21	0.29	0.63
Neuroticism										
Correlation coefficient	−0.56‡	−0.26‡	−0.32‡	−0.01	−0.24‡	−0.33‡	−0.01	−0.39‡	−0.22‡	−0.19*
*P* value	<0.001	<0.001	<0.001	0.84	<0.001	<0.001	0.81	<0.001	<0.001	0.00

LASSI, Learning and Study Strategies Inventory. *P < 0.05, †*P* < 0.01, ‡*P* < 0.001.

### Linear Regression Analysis

Multiple linear regression analysis was performed to investigate the effects of the five personality traits on the 10 LASSI scores after adjusting for sex and preclinical/clinical years. The T scores of the five personality traits were categorized into five scales and used as continuous predictors with these five scales (very high with T score > 65, high with T score 56–65, average with T score 45–55, low with T score 35–44, very low with T score < 35).

The predicted changes in the LASSI scores in relation to changes in personality traits were expressed in beta estimates and are summarized in Table 4. Increasing one scale of T scores in personality traits (e.g., moving 1 step up in the scale from low to average or from average to high increases the likelihood of using the learning strategy) predicted such changes in the LASSI scores. For example, a student who scores high on Openness (T score 56–65) would be predicted to have an 8.40 increase in their Information Processing (INP) score compared with a student who scores average in Openness (T score 45–55). This principle also applies to the effect of sex (males in comparison to females) as well as the effect of a student’s progression to the following year, which are also exhibited in Table 4.

**Table 4.** Summary of beta estimates from significant predictors associated with LASSI scores

	ANX	ATT	CON	INP	MOT	SMI	SFT	TST	TMT	UAR
Increase of 1 scale in Openness (e.g., moving 1 step up in the scale from low to average or from average to high increases the likelihood of using the learning strategy)										
Beta	3.13*	0.43	0.68	8.40‡	0.36	3.75*	3.66*	2.45	−1.92	0.30
*P* value	0.03	0.76	0.63	<0.001	0.80	0.02	0.02	0.11	0.17	0.84
Increase of 1 scale in Conscientiousness										
Beta	0.16	7.59‡	13.19‡	5.78†	18.49‡	6.34‡	8.34‡	8.45‡	16.68‡	0.94
*P* value	0.90	<0.001	<0.001	0.001	<0.001	<0.001	<0.001	<0.001	<0.001	0.49
Increase of 1 scale in Extraversion										
Beta	−0.10	4.34†	0.63	5.13†	−1.34	1.61	1.23	−0.92	0.66	5.77‡
*P* value	0.93	0.001	0.62	0.001	0.30	0.28	0.40	0.51	0.61	<0.001
Increase of 1 scale in Agreeableness										
Beta	0.50	7.53‡	1.21	−0.09	−0.79	0.93	−0.39	1.61	−2.33	0.04
*P* value	0.69	<0.001	0.34	0.95	0.54	0.52	0.79	0.24	0.07	0.98
Increase of 1 scale in Neuroticism										
Beta	−16.74‡	−3.58*	−4.99†	3.09	−2.33	−7.55‡	2.19	−9.00‡	−0.98	−2.52
*P* value	<0.001	0.02	0.001	0.08	0.14	<0.001	0.22	<0.001	0.52	0.11
Males compared to females										
Beta	10.18†	−14.21‡	−1.77	0.92	1.24	−4.39	−3.00	0.57	−4.50	−6.74*
*P* value	0.001	<0.001	0.56	0.79	0.69	0.21	0.39	0.86	0.14	0.03
Progression from one year to the next										
Beta	7.02‡	−5.14†	−2.02	0.53	2.24	4.73†	1.76	10.42‡	1.54	−0.68
*P* value	<0.001	0.001	0.17	0.75	0.13	0.005	0.29	<0.001	0.29	0.65
Adjusted *R*^2^	0.37	0.29	0.31	0.15	0.42	0.18	0.10	0.32	0.38	0.10

LASSI, Learning and Study Strategies Inventory. Beta estimates were selected based on *P < 0.05, †*P* < 0.01, ‡*P* < 0.001.

Among the five personality traits, Conscientiousness was the best predictor and was correlated with eight LASSI scores (Attitude, Concentration, Information Processing, Motivation, Selecting Main Ideas, Self-Testing, Test Strategies, and Time Management) after adjusting for other covariates. Neuroticism was the second-best predictor, predicting five LASSI scores (Anxiety, Attitude, Concentration, Selecting Main Ideas, and Test Strategies). This was followed by Openness, which predicted four LASSI scores (Anxiety, Information Processing, Selecting Main Ideas, and Self-Testing). Extraversion predicted three LASSI scores (Attitudes, Information Processing, and Using Academic Resources), and Agreeableness predicted only Attitudes.

Among the 10 regression models, the one with highest adjusted *R*^2^ (0.42) was the association between Conscientiousness and Motivation. The multiple variance inflation factor (VIF) of all predictors was lower than 10, which suggested that there was no multicollinearity issue in the 10 multiple linear regression models.

Compared with females, males had higher Anxiety scores (beta = 10.18), lower Attitude scores (beta = −14.21), and lower scores of Using Academic Resources (beta = −14.21). In addition, more preclinical/clinical training experience was associated with higher Anxiety scores (beta = 7.02), higher Selecting Main Ideas scores (beta = 4.73), higher Test Strategies scores (beta = 10.42), and lower Attitude scores (beta = −5.14).

## DISCUSSION

Employing personality sciences in the context of mentoring students is commonly faced with skepticism and concern that the risks of stereotyping outweigh the benefits of using them. Therefore, commonly many educators disregard personality and eliminate the use of its tools from their mentorship practice. One important reason for this notion is the misconception that personality traits change over time, when in fact research has shown that they are fairly stable across an individual life span (40). Furthermore, studies have shown that, despite their stability, individuals do benefit from optimizing the strengths and weaknesses of their traits, which are different in varying contexts. Therefore, mentors who help students early in their careers identify their traits, and the potential behaviors associated with these traits, offer their students an impactful advantage that enhances all elements of their life moving forward. This is especially true when mentors empower their students to optimize their traits for different contexts. Therefore, interventions utilized by the mentors would have a positive effect on the students despite the fact that students spend few years in medical school, as evident in many applications published in the literature (41–43).

Students’ behaviors in academic contexts are impacted by personality traits. Our results in this study are an example of such an impact, as they revealed that students’ personality traits predicted the selection of learning strategies.

### Conscientiousness

Conscientiousness was found to have a positive correlation with seven learning strategies and was the most important predictor of learning strategies that our medical students are likely to employ. This was especially true for the learning strategies related to the Self-Regulation component: Time Management, Concentration, and Self-Testing. This is most likely because students who score high on the Conscientiousness scale have a proclivity to being industrious, organized, dutiful, and achievement strivers (44).

Motivation had the highest correlation with Conscientiousness among the 10 learning strategies. This relates to the fact that conscientious students employ a strategic learning approach when studying (45). This relationship can be explained by the fact that students who score high on Conscientiousness are intuitively goal oriented with high aspirations and a strong sense of purpose in life, which make them motivated to learn to achieve their goals.

The second highest correlation was with Time Management. Time Management helps students take more responsibility for their behaviors through setting realistic scheduled plans to deal with distractions, competing goals (relevant or irrelevant to their academic outcomes), and procrastination. Since procrastination has been found to be common among those who are not conscientious, it is important to identify these students early on and offer them individualized mentorship (46). The present findings can be further explained by knowing that conscientious students are goal oriented and achievement strivers.

Finally, there was a positive correlation between Conscientiousness and Concentration. Previously, Concentration was shown to predict medical students’ performance in the USMLE Step 1 (34), National Board of Chiropractic Examiners (47), and overall preclinical biomedical sciences (32). This means that students who score higher on Concentration are more likely to perform better in medical school examinations and in licensing examinations. This is important information for mentors as it helps them identify medical students with potential concentration problems and allows for early interventions targeted toward mentees’ Concentration skills. The findings may be explained by the fact that conscientious students are goal oriented, which helps them maintain focus on their academic tasks.

Since conscientious students were also considered achievement oriented, persistent, and self-disciplined, which are important characteristics of academic success, it was fair to expect that possessing Conscientiousness goes hand in hand with the following: positive attitude toward college education (Attitude), the ability to select key concepts from study material (Selecting Main Ideas), as well as practicing information retrieval in a manner effective for formal medical college exams (Test Strategies), which this study has also demonstrated.

The findings show that enhancing Conscientiousness can lead to an improvement in learning strategies for medical students. This has great implications for the practice of academic mentorship. Thus, evaluating Conscientiousness should be the starting point for academic guidance, as it would be expected to yield the greatest effect on students’ use of learning strategies. This is especially important when identifying less conscientious students. In such cases, mentors can evaluate the different Conscientiousness-related learning strategies, work with their mentees on improving them, and then track their progress in developing these study strategies. This helps mentors to better individualize their mentorship and fit the needs of their students.

### Neuroticism

Neuroticism was found to be correlated with four learning strategies. Like Conscientiousness, the effect of Neuroticism spans all three components of the learning strategies, but the correlation here was negative with all of them. The highest was with Anxiety scores.

This finding was not surprising, as anxiety has long been considered a primary facet of neuroticism (37), and this study shows its negative effects on many learning strategies. Neurotic students are prone to constant worries and frequently experience negative emotions. Therefore, they may express disengaging coping mechanisms, which in turn can lead students to fail at organizing and categorizing their learning into meaningful units. Because of this, anxious students can become less motivated and persistent in their approach to learning (48–50). This is important information for mentors, as they need to evaluate the anxiety of their mentees and help them develop the necessary skills needed to reduce the burden of stress and possible burnout that may result (51).

Moreover, the findings showed that the following study strategies were all negatively correlated with Neuroticism: Test Strategies, Selecting Main Ideas, and Concentration. This means that students who score high on the Neuroticism domain are more likely to have problems maintaining focus and weaker testing skills (the ability to properly prepare for tests of different types and items) when it comes to exam taking or preparation.

These negative influences may be explained by the fact such students tend to have negative thoughts, such as self-doubt, which may cause cognitive learning interference during encoding and storage efforts that occur during studying and test preparation, leading eventually to a less solid formation of knowledge (52). Therefore, mentors of these students should screen these learning strategies individually to provide students with additional resources and training. For example, mentors could encourage such students to learn some techniques that improve Concentration skills (34) as well as advise students to solve practice questions and take mock exams to better develop their Test Strategies (53).

Students scoring high on the Neuroticism scale are also predicted to have poorer ability in Selecting Main Ideas and key concepts, leaving them lost when dealing with large amounts of medical material. Therefore, mentors could advise such students to organize the information they study with methods such as summaries or mind maps. This would help medical students differentiate key concepts from supporting details, improving their learning and information retention (53, 54).

Despite the many benefits of identifying students with high score in Neuroticism, some educators and mentors might be worried about potential negative consequences associated with a student being labeled as neurotic. These concerns are primarily due to a misconception that having high Neuroticism is always detrimental, whereas in reality Neuroticism’s negative effects are contextual. This means that high Neuroticism could be harmful in some contexts, some structured academic environments for example, but it also can be beneficial in other contexts such as a busy work environment for instance, or even for some health dimensions (43, 55, 56). Addressing such misconceptions and destigmatizing Neuroticism are examples highlighting the importance of introducing and training medical educators in personality sciences and their potential uses to help their students. It was our hope through this project that we can contribute to raising awareness among mentors to help counter such misconceptions. Having said that, colleges are advised to develop guidelines that govern the usage and disclosure of personality data to mitigate any minor but potential harms that can result from identifying these scores.

### Extraversion, Openness, and Agreeableness

Extraversion was positively correlated with Using Academic Resources and Information Processing. This could be explained by the tendency of extroverts to socialize, which helps develop their Information Processing through communication and extended exchange of experiences. Their sociability also makes it easier for them to seek help from others, which contributes to better Use of Academic Resources as well as making such students efficient learners (57, 58). This has an important application when mentoring extroverted students, as encouraging them to use more group and interactive learning activities could possibly improve their Information Processing and Using Academic Resources skills (11).

Moreover, Extraversion was also positively correlated with Attitude scores, meaning that extroverts exhibit a more productive attitude toward learning. This may be explained by the fact that extroverts usually experience more positive emotions because of extraversion’s relationship with dopaminergic brain functions, which are essentially involved in reward processing, learning, decision-making, and risk assessment (59). This may contribute to a better attitude toward learning, especially in group settings. Therefore, mentors can maximize the benefits of these attributes through advising such students to capitalize on the advantages of study groups and other similar group learning activities.

Regarding Openness, Information Processing was positively correlated with Openness. This was consistent with the literature, since students who score high on Openness tend to utilize more higher-order cognitive skills such as elaborative processing, critical thinking, and constructive learning approaches, leading to deep learning (16, 45, 57, 60, 61).

However, we expected Information Processing to have stronger correlations and predictive values, which was not the case. This could possibly be attributed to the highly structured environment of medical schools and the issue of medical curriculum overload (62). This could limit students with high Openness from harnessing their natural abilities for imagination and critical and creative thinking (45). Mentors may help these students through exposing them to opportunities where they can freely tap into their creativity, problem-solving, and reflection to enhance their learning. For example, mentors could involve these students in more problem-based learning or case discussion activities.

Agreeableness had a positive correlation with Attitude scores. This may be explained by the compassionate nature of Agreeable students, which makes them perceive studying medicine positively, which was reflected in their Attitude scores. Mentors can expect less agreeable students to express negative attitudes toward learning and not to see the value of the different learning activities they undergo. Thus, mentors can play an important role in explaining the relevance of the curriculum components and the different teaching activities to improve their attitudes toward learning.

Despite the findings, Agreeableness should not be excluded from personal development mentorship, as many physician roles are related to the affective domain of learning, especially in community-based medical education (63), and such roles are essentially related to the Agreeableness trait. These include, but are not limited to, communication skills, professionalism, empathy, respect, and multidisciplinary teamwork.

### Sex Differences

Findings showed that personality traits similarly affect how students select learning strategies in both sexes. The only exception was related to Anxiety, Attitude, and Using Academic Resources. Since Anxiety was highly correlated and predicted by Neuroticism, and females exhibit higher Neuroticism generally, they have a tendency to be more anxious. This finding was not surprising and consistent with the literature (64, 65). Similarly, females seem to have higher scores in Attitude toward learning and Using Academic Resources compared with males, and this could be explained by the reported correlation between females and the traits of Agreeableness and Extraversion (57), both of which are related to Attitude and Using Academic Resources. Having said that, further studies are needed to explore this matter.

### Differences Between Preclinical and Clinical Year Students

There was only a difference in the following four learning strategies: Test Strategies, Anxiety, Selecting Main Ideas, and Attitude. As students progress, their scores in the first three strategies improve. This could be because they gain more experience in handling stress, coping with exams, and identifying key concepts.

Furthermore, as students progress, their scores in Attitude toward learning decrease. This could be because they realize the magnitude of challenges that lie ahead of them in terms of residency training and the demands of medical practice. These findings indicate the importance of considering the effects of personality traits on academic skills in all years. Thus, early personality-based mentorship interventions in preclinical years could be beneficial at all academic levels and especially produce higher impact in the clinical years, because of the compound effects of such early efforts targeted at trait optimization.

### Limitations

This was a cross-sectional project, and no causal relationships can be inferred (66). Second, the NEO-FFI-3 used was the short version of the personality questionnaire, and it only measures the general characteristics of the big five domains. These domains of personality are considered broad traits, with each consisting of lower-level aspects (67) and a wider range of lower-level facets (37) that are not individually analyzed in this study. These would provide a more detailed profile of students’ traits. Investigating the use of the longer version of the questionnaire (NEO-PI-3) in future studies is an area to consider. Third, it has been reported that the relationship between self-view, also known as self-concept, and learning strategies was reciprocal (68). Students’ self-concept, which is potentially affected by their personality traits, could be a potential confounder. However, further studies are needed to explore the effects of personality-based perception on students’ self-evaluation of their learning strategies. Fourth, despite considering Pearson’s correlations in the range of 0.3–0.5 as potentially meaningful in the context of this study, they should not be treated as definite predictors. This is because >50% of the variance in our outcomes was unexplained. Thus, mentors must be careful not to overstate the importance of being “neurotic” or “conscientious” as being definitively a predictive trait. Finally, we did not investigate how our findings relate to performance outcomes. We plan to explore this relationship in upcoming studies where we account for measuring performance in different domains (e.g., affective outcomes) and exploring their relationships with our findings.

### Conclusions

All personality domains have an influence on at least one learning strategy, especially Conscientiousness and Neuroticism. Through the predictive quantification that this study provides, mentors have a practical, objective, and accurate means to predict potential shortcomings among their mentees that result from their trait tendencies. Thus, they can plan timely interventions and track their effectiveness. This action research aspires to inspire a more holistic mentorship approach where educators consider personality traits and learning strategy selection. Thus, we recommend incorporation of personality traits and learning strategy assessments in the formal training of mentors, academic coaches, clinical educators and curriculum developers.

## GRANTS

The authors acknowledge the generous funding provided by Alfaisal University’s Internal Research Grant (IRG).

## DISCLOSURES

No conflicts of interest, financial or otherwise, are declared by the authors.

## AUTHOR CONTRIBUTIONS

R.A.A., A.M.A.M., S.M., and H.S.S. conceived and designed research; R.A.A. and M.A.B.A. performed experiments; R.A.A. analyzed data; R.A.A. and A.M.A.M. interpreted results of experiments; R.A.A. prepared figures; R.A.A., A.M.A.M., M.A.B.A., S.M., and H.S.S. drafted manuscript; R.A.A., A.M.A.M., M.A.B.A., S.M., and H.S.S. edited and revised manuscript; R.A.A., A.M.A.M., M.A.B.A., S.M., and H.S.S. approved final version of manuscript.
